# Molecular Identification and Subtype Analysis of *Blastocystis* sp. Isolates from Wild Mussels (*Mytilus edulis*) in Northern France

**DOI:** 10.3390/microorganisms12040710

**Published:** 2024-03-30

**Authors:** Manon Ryckman, Nausicaa Gantois, Ruben Garcia Dominguez, Jeremy Desramaut, Luen-Luen Li, Gaël Even, Christophe Audebert, Damien Paul Devos, Magali Chabé, Gabriela Certad, Sébastien Monchy, Eric Viscogliosi

**Affiliations:** 1 CNRS, Inserm, CHU Lille, Institut Pasteur de Lille, U1019–UMR 9017–CIIL–Centre d’Infection et d’Immunité de Lille, University of Lille, F-59000 Lille, France; manon.ryckman@etu.univ-littoral.fr (M.R.); nausicaa.gantois@pasteur-lille.fr (N.G.); jeremy.desramaut@pasteur-lille.fr (J.D.); damienpdevos@gmail.com (D.P.D.); magali.chabe@univ-lille.fr (M.C.); gabriela.certad@pasteur-lille.fr (G.C.); 2 Université du Littoral Côte d’Opale, CNRS, University Lille, UMR 8187, LOG, Laboratoire d’Océanologie et de Géosciences, F-62930 Wimereux, France; luen-luen.li@univ-littoral.fr (L.-L.L.); sebastien.monchy@univ-littoral.fr (S.M.); 3 Centro Andaluz de Biología del Desarrollo, CSIC, Universidad Pablo de Olavide, 41013 Sevilla, Spain; ruben.garcia.d93@gmail.com; 4 GD Biotech—Gènes Diffusion, F-59000 Lille, France; g.even@genesdiffusion.com (G.E.); c.audebert@genesdiffusion.com (C.A.); 5 PEGASE-Biosciences (Plateforme d’Expertises Génomiques Appliquées aux Sciences Expérimentales), Institut Pasteur de Lille, F-59000 Lille, France; 6 Délégation à la Recherche Clinique et à l’Innovation, Groupement des Hôpitaux de l’Institut Catholique de Lille, F-59000 Lille, France

**Keywords:** *Blastocystis* sp., mussels, intestinal parasites, molecular epidemiology, transmission sources, zoonosis, fecal contamination

## Abstract

*Blastocystis* sp. is the most common single-celled eukaryote colonizing the human gastrointestinal tract worldwide. Because of the proven zoonotic potential of this protozoan, sustained research is therefore focused on identifying various reservoirs of transmission to humans, and in particular animal sources. Numerous groups of animals are considered to be such reservoirs due to their handling or consumption. However, some of them, including mollusks, remain underexplored. Therefore, a molecular epidemiological survey conducted in wild mussels was carried out in Northern France (Hauts-de-France region) to evaluate the frequency and subtypes (STs) distribution of *Blastocystis* sp. in these bivalve mollusks. For this purpose, 100 mussels (*Mytilus edulis*) were randomly collected in two sampling sites (Wimereux and Dannes) located in the vicinity of Boulogne-sur-Mer. The gills and gastrointestinal tract of each mussel were screened for the presence of *Blastocystis* sp. by real-time polymerase chain reaction (qPCR) assay followed by direct sequencing of positive PCR products and subtyping through phylogenetic analysis. In parallel, sequences of potential representative *Blastocystis* sp. isolates that were previously obtained from temporal surveys of seawater samples at marine stations offshore of Wimereux were integrated in the present analysis. By taking into account the qPCR results from all mussels, the overall prevalence of the parasite was shown to reach 62.0%. In total, more than 55% of the positive samples presented mixed infections. In the remaining mussel samples with a single sequence, various STs including ST3, ST7, ST14, ST23, ST26 and ST44 were reported with varying frequencies. Such distribution of STs coupled with the absence of a predominant ST specific to these bivalves strongly suggested that mussels might not be natural hosts of *Blastocystis* sp. and might rather be carriers of parasite isolates from both human and animal (bovid and birds) waste. These data from mussels together with the molecular identification of isolates from marine stations were subsequently discussed along with the local geographical context in order to clarify the circulation of this protozoan in this area. The identification of human and animal STs of *Blastocystis* sp. in mussels emphasized the active circulation of this protozoan in mollusks and suggested a significant environmental contamination of fecal origin. This study has provided new insights into the host/carrier range and transmission of *Blastocystis* sp. and emphasized its potential as an effective *sentinel species* for water quality and environmental *contamination*.

## 1. Introduction

*Blastocystis* sp. is a cosmopolitan protozoan belonging to the Stramenopile group and colonizing the intestines of humans [1,2,3,4], as well as those of a wide range of animals from non-human primates and birds to fish and insects [5,6,7,8]. Because of its predominant transmission via the fecal–oral route, its prevalence is particularly alarming in developing countries, where poor sanitary and hygiene conditions and unavailability of effective water treatment facilitate the circulation of intestinal parasites [9]. Consequently, its frequency may well exceed 50% in human cohorts, as evidenced in recent epidemiological surveys conducted, for instance, in African countries [10,11,12].

A large majority of individuals harboring *Blastocystis* sp. are asymptomatic, which has long raised questions about the pathogenicity of this protozoan [13,14,15]. However, clinical cases of colonized patients have highlighted the link between the presence of the parasite and the development of gastrointestinal disorders and even urticaria [16,17,18]. In addition, the pathogenicity of certain isolates has been demonstrated in vitro and/or in vivo, allowing the identification of virulence factors leading to damaging effects on the host intestinal epithelium [19,20]. To supplement these data, the impact of *Blastocystis* sp. on the host gut bacterial community has been demonstrated to be beneficial or deleterious depending on the genetic differences of the parasite isolates [21,22,23].

Based on small subunit (SSU) rDNA gene sequences comparison, this protozoan exhibits a wide intra-genus genetic diversity, with no fewer than 42 so-called subtypes (STs) currently identified as valid, with two of them (ST10 and ST43) divided into two subgroups (ST10a/ST10b and ST43a/ST43b) [24,25,26,27,28,29,30]. Interestingly, more than 90% of the subtyped isolates in humans belong to ST1 and to ST4, likely related to widespread human-to-human transmission [11,31,32]. Most of the others STs were mainly identified in various animal groups as, for instance, ST6/ST7, ST10/ST14 and ST5 are predominantly found in birds, bovids and pigs, respectively [2,5,6]. The presence of these latter STs in the human population therefore strongly suggest zoonotic transmission that was fully evidenced through the finding of the same genetic variants circulating simultaneously among animals and their handlers as well as in non-human primates zookeepers [33,34] and staff of commercial intensive piggeries [35] and poultry slaughterhouses [36].

Despite its proven zoonotic potential, *Blastocystis* sp. remains poorly investigated in various animal groups nevertheless regarded at risk for humans, as, for instance, mollusks and more globally, shellfish. In particular, mussels can represent potential reservoirs of transmission through their handling and most importantly, their consumption raw or undercooked. Moreover, as filter feeders, these bivalves have the capacity to filter over 2 L of water/h/shellfish through their bodies to grab phytoplankton and other particles while also accumulating potential contaminants such as bacteria and waterborne protozoa [37]. To our knowledge, only two studies are available in the literature regarding the identification of *Blastocystis* sp. in mussels. The first conducted in Poland identified the protozoa by microscopy with a low prevalence in tissues of freshwater duck mussels [38], while the second carried out in Chile and using molecular detection methods, highlighted a significant frequency of *Blastocystis* sp. in batches of Chalga mussels [39]. In the face of such limited data and the strong interest in identifying animal reservoirs of human infection, the aim of the present study was therefore to determine the frequency and ST distribution of *Blastocystis* sp. in mollusks by screening seawater mussels collected from the coastline of the Hauts-de-France region (Boulogne-sur-Mer area) and then, based on these data, to assess the potential risk of zoonotic transmission of the parasite from these bivalves.

## 2. Materials and Methods

### 2.1. Mussels Sampling Areas

**Figure 1:**
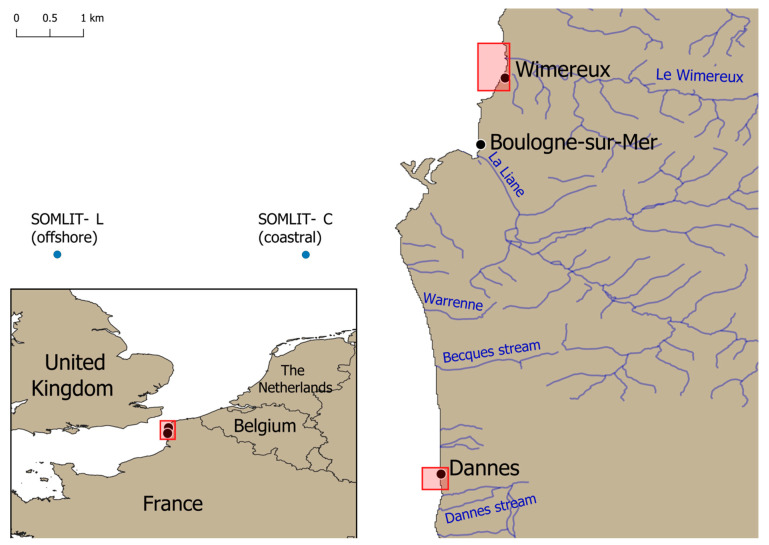
Location of the two mussel harvesting areas on the foreshores of Wimereux and Dannes (red boxes) and the two SOMLIT stations (blue dots).

This survey was conducted in early February 2023 on the eastern English Channel (EEC) coastline near the city of Boulogne-sur-Mer in the Hauts de France region, located in the North of France, about a hundred kilometers from the Belgian border. A total of 100 wild mussels (*Mytilus edulis*) were manually and randomly collected at the foreshores of two different sampling sites, Wimereux and Dannes, approximately thirty kilometers apart (Figure 1). Briefly, 50 wild mussels were gathered at low tide along the Wimereux coast at different sampling points on rocks or other supports extending over around 500 m. This collection zone was precisely located at the Wimereux river mouth. Briefly, this river flows through the beach resort of Wimereux and is supplied upstream by several streams, some of which are close to dairy cattle farms. The remaining 50 mussels were collected along the coast about two kilometers from the city of Dannes over an area of around 200 m on various supports. This area hosting mussel breeding farms is less urbanized than Wimereux with a few small streams flowing into the sea.

### 2.2. Processing of Mussels

Each mussel was individually wrapped in foil then transported in a freezer bag to the laboratory, where they were stored at 4 °C until dissection. The wild mussels were subsequently removed from their individual packaging before being weighed (13.3 g ± 3.07 g) and measured (length 4.95 cm ± 0.35 cm and width 2.11 cm ± 0.2 cm), then washed externally with sterile distilled water before being opened by a scalpel section at the level of the posterior adductor muscle. The gills and the gastrointestinal tract were carefully excised from each mollusk, placed individually in 2 mL Eppendorf tubes on ice then dilacerated using sterile tips before storage at −20 °C.

### 2.3. DNA Extraction and PCR Identification of Blastocystis sp.

DNA extraction from approximately 250 mg of dilacerated samples of gills or gastrointestinal tracts was performed using the NucleoSpin 96 Soil kit (Macherey-Nagel GmbH & Co KG, Düren, Germany) following instructions recommended by the manufacturer. The DNA was eluted in 100 µL of elution buffer provided in the kit, then stored at −20 °C until use. Briefly, purified DNA (2 µL) was amplified by real-time PCR (qPCR) assay targeting a domain of approximately 300 bp of the SSU rDNA gene using the primer pair sense BL18SPPF1/antisense BL18SR2PP specific to the *Blastocystis* genus as previously described [40]. All qPCRs were performed in duplicate with positive (*Blastocystis* sp. ST8 DNA from an axenic culture) and negative (DNA replaced by water in the mixture of PCR reagents) controls. All the positive samples by qPCR were purified then sequenced on both strands using the primer pair BL18SPPF1/BL18SR2PP by the company Genoscreen (Lille, France; SANGER technology platform, 3730XL DNA analyzer). Sequence chromatograms showing double traces reflecting mixed infections (presence of at least two different *Blastocystis* sp. STs in the same sample) were obtained for a significant number of mussel samples. For these positive specimens, the STs colonizing the corresponding mollusks remained undetermined. The sequences obtained in the present study for single infections by *Blastocystis* sp. were deposited in GenBank under accession numbers PP357056–PP357090.

### 2.4. Blastocystis sp. Isolates from Seawater Samples

From the same geographical area of Boulogne-sur-mer, the diversity of planktonic eukaryotic microbes in subsurface water samples (2–3 m water depth) was previously studied monthly at two SOMLIT (French Network of Coastal Observatories) marine stations (Coastal C and Offshore L) of the EEC in 2012 and 2013 using high throughput sequencing targeting the SSU rDNA gene [41,42]. SOMLIT C and SOMLIT L stations are located 1.5 and 8 km from the coast, respectively (Figure 1). After high throughput sequences analysis and quality filtering, Operational Taxonomic Units (OTUs) were searched on the Protist Ribosomal Reference database (PR2) [43] using blastn [44] for taxonomic assignment. Potential OTUs representatives of *Blastocystis* sp. were manually verified using blastn on GenBank and integrated in the present survey. The sequences from SOMLIT eukaryote diversity survey were deposited in GenBank-SRA under the accession number SRX768577, as well as individual OTUs affiliated to *Blastocystis* sp. under accession numbers PP527163 (OTU0278), PP527163 (OTU0438) and PP527165 (OTU0680).

### 2.5. Phylogenetic Analysis and Subtyping of Blastocystis sp. Isolates

Full-length SSU rDNA gene sequences from representatives of the different STs and subgroups of *Blastocystis* sp. available at the time of the analysis were extracted from databasesGenBank to represent a reference framework. To this foundational dataset, fragments of various regions of the same molecular marker obtained from a diverse array of *Blastocystis* sp. isolates were added. This included all sequences of mussel isolates obtained in this study and four sequences (OTUs) from environmental sources (SOMLIT stations C and L) reflecting the genetic diversity of the different reads obtained by high throughput sequencing and representative of *Blastocystis* sp. This approach underlined the versatility of our phylogenetic analysis method, capable of integrating both complete sequences and specific sequence fragments, thus facilitating nuanced positioning of isolates from different origins in the phylogenetic tree. The phylogenetic analysis was conducted using MAFFT v7.490 for sequence alignment, employing the L-INS-i method for its robustness in aligning a diverse set of sequences [45]. Initial attempts to trim the alignment with TrimAl to a threshold of 0.7 were performed [46]. However, the maximum-likelihood phylogenetic analysis was conducted with the untrimmed alignments as they provided a higher resolution of the tree structure and samples placements. The reference tree was constructed with IQ-TREE using the K2P + I + G4 model, chosen to maintain consistency with the substitution model and with 1000 bootstrap replicates [47]. The tree was rooted on the cluster ST15/ST28 showing the earliest emergence within the *Blastocystis* genus in recent phylogenetic analyses [26,27,28,29]. Placement of sequences was refined with EPA-ng, a tool designed for precise phylogenetic placement [48], and the resulting data were processed and visualized using Gappa [49]. Further visualization enhancements and annotations were made using the Interactive Tree Of Life (iTOL) platform, which allowed for a more comprehensive representation of the phylogenetic relationships [50].

## 3. Results and Discussion

To our knowledge, this survey represents the most comprehensive assessment of the molecular epidemiology of *Blastocystis* sp. in mollusks, and more specifically, in mussels. In this context, 100 wild mussels were randomly collected on the French EEC coastline in two sampling zones, Wimereux and Dannes, then dissected to analyze the gills and digestive tract of each bivalve individually for the presence of *Blastocystis* sp. Of the 50 mussels sampled along the Wimereux coast, 16 (32.0%) were positive by qPCR assay in the gills, 18 (36.0%) in the digestive tract and 10 (20%) in both organs (Table 1 and Table 2). Thus, 48.0% of the mussels in this gathering area were positive for the parasite in one or both of these organs.

In Dannes, 58.0% (29/50) of mussels analyzed were found to be positive for *Blastocystis* sp. in the gills, 38.0% (19/50) in the digestive tract and 20% (10/50) in both organs, i.e., a total of 76.0% of mussels were infected from this collection site (Table 1 and Table 2). By combining the data from Dannes and Wimereux, 62.0% of the mollusks tested in this survey were thus colonized by the protozoan in one or both organs.

Consequently, the frequency of infection of mussels observed at both sites and globally in the North of France region was significantly higher than that first reported in two species of freshwater bivalves, *Anadonta anatina* and *Unio tumidus*, collected along the shoreline of Lake Malta (municipal reservoir) in the vicinity of Poznan in Poland [38]. Indeed, this protozoan was identified using direct-light microscopy in only 5.1% of the *A. anatina* specimens analyzed, predominantly in the gastrointestinal tract (75.0%) of animals, and none of the investigated samples of *U. tumidus* were colonized by *Blastocystis* sp. Nonetheless, these data have to be interpreted with caution given that conventional diagnostic methods including microscopy are known to greatly underestimate the prevalence of *Blastocystis* sp. compared to molecular approaches [40]. In addition, the parasite was also detected by PCR in marine Cholga mussels obtained from commercial stores and caught in the bay of Conception in Chile [39]. However, no prevalence data could be inferred through this study since *Blastocystis* sp. was only sought in groups of mussels and not from the individual screening of mollusks.

**Figure 2:**
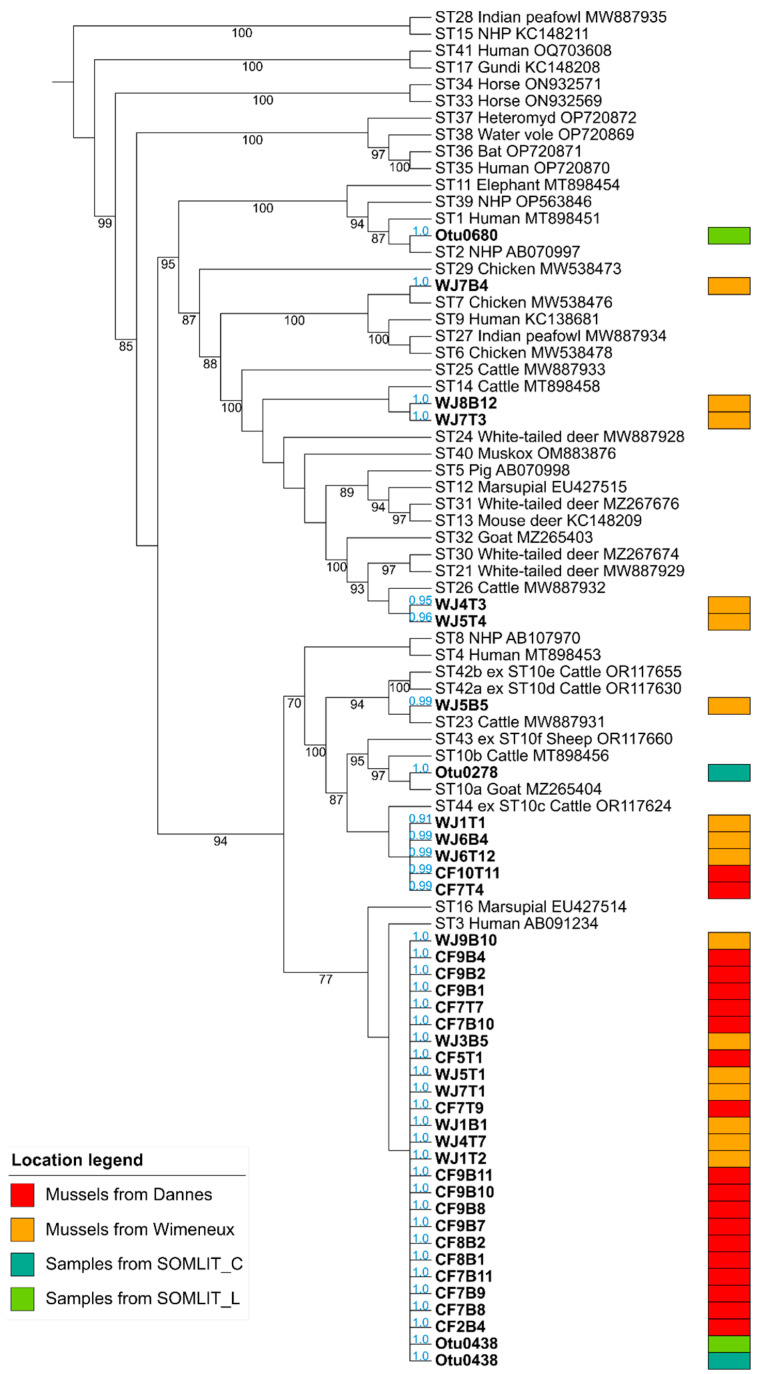
Maximum likelihood phylogenetic analysis of *Blastocystis* sp. isolates based on partial SSU rDNA gene sequences. The tree has been rooted on the reference sequences of the earliest diverging cluster ST15/ST28 within the *Blastocystis* genus. Sequences obtained in the present study (WJ, Wimereux, orange boxes; CF, Dannes, red boxes) are in bold as those corresponding to environmental samples (SOMLIT stations, blue and green boxes). Accession numbers of reference sequences of known STs are indicated. Bootstrap values are in black at the nodes of tree. Values below 70% are not indicated. Values in blue near each isolate analyzed correspond to the likelihood of each sequence to be placed in that specific node.

In a second step, the qPCR products of the 82 positive samples (both gills and intestinal tracts) obtained herein were purified, then directly sequenced. Through the analysis of the corresponding sequence chromatograms, more than half of them exhibiting double traces (47/82, 57.3%) reflecting mixed infections, meaning more than one ST present in the same sample (Table 2). This proportion of mixed infections in the positive samples analyzed was of the same order of importance for mussels collected at Wimereux (18/34, 52.9%) as for those sampled at Dannes (29/48, 60.4%). High proportions of mixed infections have also been reported in numerous other animal groups, as recently described [5,29,51], definitely related to multiple sources of contamination in their respective environment. The remaining 35 chromatograms reflected single infections in either gills or intestinal tract of mussels from the two sampling points. The corresponding sequences were subsequently included in a large phylogenetic analysis allowing for the successful and definite subtyping of all the mussels isolates according to likelihood values (0.91 to 1.0) (Figure 2). The few isolates identified from SOMLIT marine stations (Table 3) were also integrated in the same tree reconstruction and subtyped.

A large genetic diversity of *Blastocystis* sp. isolates colonizing mussels from Wimereux was highlighted with the identification of six STs circulating in this sampling area, with a predominance of ST3 (7/16, 43.8%) followed by ST44 (3/16, 18.8%), ST14 (2/16, 12.5%), ST26 (2/16, 12.5%), ST7 (1/16, 6.2%) and ST23 (1/16, 6.2%) (Table 2 and Figure 2). In contrast, only two STs have been identified in mussels from the site of Dannes, namely ST3, which accounted for the vast majority of isolates (17/19, 89.5%), and ST44 (2/19, 10.5%). To complement these subtyping data, sequences of seawater isolates collected at SOMLIT stations were identified as belonging to ST2 (SOMLIT L), ST3 (SOMLIT C and SOMLIT L) and ST10a (SOMLIT C) (Table 3). Numerous mussels sampled in Wimereux and Dannes contained various combinations of STs, the vast majority of which remain undetermined due to the failure to identify STs composing mixed infections (Table 2). However, the combination of ST3 in gills (WJ1B1) and ST44 in the gastrointestinal tract (WJ1T1) was recorded in the Wimereux mussel WJ1-1 and the same isolate of ST3 was reported in both the gills (CF7B9) and intestinal tract (CF7T9) of the Dannes mussel CF7-9.

All these data focused on the frequency and distribution of STs of *Blastocystis* sp. in mussels together with the molecular identification of isolates from marine stations required to be analyzed in a local geographical context considering all environmental parameters in order to clarify the sources and circulation of the protozoan. Regarding mussels collected in Wimereux, although the frequency of the parasite was lower than that reported in Dannes, the diversity of STs identified from single infection data was nevertheless greater, with the presence of six STs colonizing the bivalves. ST3, which was predominant, is also by far the commonest ST in the human population worldwide [1,2,3,4,11,31], and therefore its high occurrence in the mollusks was most likely linked to contamination of seawater by human feces. Interestingly, ST3 was also the only ST identified in groups of Cholga mussels in Chile [39]. Still, at the Wimereux sampling site, the presence of an ST7 isolate considered as an avian ST because of its predominance in birds [2,5,36] and of representatives of ST14, ST23, ST26 and ST44 sharing the frequent colonization of domestic cattle and small ruminants [5,28,52] could be explained by contact of bivalves with seawater contaminated with bird droppings and bovid fecal material, respectively. At the Dannes collection point, only two STs, ST3 and ST44, were reported. As detailed above, the presence of these STs was undoubtedly linked to contact between the mussels and seawater polluted with human (for ST3) and bovid (for ST44) waste. Therefore, the wide diversity of STs identified in these mollusks, coupled with the absence of a predominant ST specific to these bivalves, suggests that mussels might not represent natural hosts of *Blastocystis* sp., and that they act rather as carriers of parasite isolates from human and others animal groups. However, further research is needed to study if the parasite is able to multiply in this host through histological analysis of digestive tissues as investigated in fish [7].

The frequency of the parasite and the genetic diversity of isolates in mussels were also convincing signs of high levels of fecal contamination through multiple pollution reservoirs. Around Wimereux, a number of farms are primarily specialized in raising Prim Holstein dairy cattle, potentially colonized by STs well adapted to persistent colonization of cattle including ST14, ST23, ST26 and ST44 among others [5,28,52]. Interestingly, a recent epidemiological survey carried out on cow farms in the North of France showed that more than half of the animals tested were colonized by the parasite, with a very large predominance of the host-adapted ST14 and ST10 [53] also found in mussels and marine stations sampled in the present survey. Moreover, several streams feeding into the Wimereux flow close to these premises, and potential discharges and runoff from these farms can carry bovid STs of the parasite to the Wimereux river and then to its mouth, where the mussels were collected. Strikingly, an inventory commissioned in the urban area of Wimereux in 2017 reported around thirty wastewater and rainwater effluents together with discharges of an undetermined nature [54], facilitating the transmission of *Blastocystis* sp. isolates of human or animal origin. Moreover, the impact of effluents from the sewage treatment plant of Wimereux on mussels remains unknown at this time.

In Dannes, the frequency of mussels colonized by the protozoan was surprisingly higher than in Wimereux, despite the facts that only five streams representing potential waste discharges were localized in this zone [55], and that this town is located relatively far from the coast. However, open year-round campsites on the seafront could represent potential sources of contamination by the human *Blastocystis* sp. ST3, which was reported to be highly predominant in infected mussels from Dannes. Moreover, this zone is a known mussel-growing area with a very high density of mussels and consequently, self-contamination of mollusks with human ST3 cannot be excluded. This zone could also be influenced by various wastewater treatment plants located near the town and at the Canche estuary located 4.5 km south of the city. On the other hand, directed movements of seawater could also play a potential role in the spread of the parasite from Wimereux to Dannes, since the down welling current is oriented towards the south and could maintain contaminants including *Blastocystis* sp. at the coast [54].

A mathematical model taking into account flow rate and contaminant flux in number of *Escherichia coli* per day at several sites along the Boulogne-sur-Mer coastline was previously developed [56] and revealed a maximum level of contamination near Wimereux Bay. This pollution also extended offshore, undoubtedly explaining the identification of *Blastocystis* sp. in seawater samples analyzed in 2012–2013 at depths of 2 or 3 m at SOMLIT stations located 1.5 and even 8 km from Wimereux. The STs identified corresponded to STs mainly adapted to human (ST1 and ST3) and bovine (ST10a) hosts like those characterized in mussels, again in relation to fecal contamination from these potential hosts of the parasites. Interestingly, the number of reads corresponding to the OTUs 0278 and 0438 from SOMLIT C was much higher in summer than in winter (Table 1), emphasizing the higher prevalence of *Blastocystis* sp. ST3 and ST10a along the coast in summer. Wimereux is a sea resort town whose population doubles in summer and therefore, the higher frequency of ST3 at this time of the year was likely attributable to the increase in population and recreational activities (anthropic pressure). This was perfectly evidenced, for example, in the case of rivers in Malaysia, where the occurrence of *Blastocystis* sp. was significantly boosted during holidays, in association with the water recreational activities of locals and visitors [57]. As regards the much higher number of ST10a isolates collected at SOMLIT C in summer than in winter, the fact that animals are grazing in the fields at this time of year and are not confined to their barns could clearly facilitate the dissemination of the parasite in the environment through livestock effluents. To complete the picture in the Boulogne-sur-Mer area, a recent study also identified *Blastocystis* sp. in marine mammals (common porpoise, common seal and sperm whale) stranded along the coastline and in edible marine fish (herring, whiting and mackerel) caught in the EEC, thus attesting to the widespread presence and active circulation of the parasite in seawater and consequently in marine animal groups living in this zone [7].

With regard to the significant prevalence of *Blastocystis* sp. reported herein in mussels, the question of the potential impact on human health through the consumption of infected mollusks was clearly raised. However, although these mussels harbor numerous STs potentially transmissible to humans, the health risk seems very moderate, since mussels are usually eaten well cooked in France. In addition, before commercialization, regulation imposed a 48 h depuration of mussel in purified water. This process aimed at using the natural filtration of mussels to eliminate the fecal coliforms they had accumulated. However, mussel depuration efficiency for *Blastocystis* sp. remains to be clarified. Moreover, further studies are also needed to assess the possible transmission of the protozoan to workers handling mussels, whether mussel farmers and staff of fishmongers and supermarkets.

## 4. Conclusions

To the best of our knowledge, this survey represents the largest molecular study regarding the prevalence of *Blastocystis* sp. in mollusks and more precisely in mussels and therefore, improves our understanding of the epidemiology of this protozoan in yet under investigated animal groups. For a long time, mussels have been widely used for sanitary assessment of water quality for their ability to act as natural concentration systems of contaminants. Consistently, our study conducted on the coasts of the Boulogne-sur-Mer area highlighted an environmental contamination of fecal origin reflected by the high prevalence of *Blastocystis* sp. in the mollusks. These data also confirmed the status of this protozoan as an effective *sentinel* species for water quality and environmental contamination. Based on the various STs of the protozoan identified in mussels which very likely do not represent natural hosts of the parasite, different origin and transmission patterns of *Blastocystis* sp. isolates were proposed to explain their presence in mollusks. However, to confirm these scenarios, as yet hypothetical, a One Health approach has to be conducted in this geographical area by screening throughout the year human, animal (particularly mussels and bovid), and environmental (streams and various discharges and effluents) samples. Such a survey will also enable us to clarify the seasonal impact on the circulation of the parasite and identify the various sources of contamination for the human population and mollusks. In addition, these data also stressed the importance of expanding the screening of additional mollusks in order to complete the epidemiology of *Blastocystis* sp. This includes oysters and cockles which, unlike mussels, are usually eaten raw or lightly cooked. The consumption of these latter shellfish could thus represent a real risk in human health, especially if these filter feeders are harvested from polluted areas.

## Figures and Tables

**Table 1:** Frequency of *Blastocystis* sp. in mussels by collection site and by organ.

Collection Site	Gills	Gastrointestinal Tract	Both Organs	Infected Mussels (Gills and/or Gastrointestinal Tract)
Wimereux	16/50 (32.0%)	18/50 (36.0%)	10/50 (20.0%)	24/50 (48.0%)
Dannes	29/50 (58.0%)	19/50 (38.0%)	10/50 (20%)	38/50 (76.0%)
Total	45/100 (45.0%)	37/100 (37.0%)	19/100 (19.0%)	62/100 (62.0%)

**Table 2:** Mussels positive for *Blastocystis* sp. by qPCR in the gills and/or gastrointestinal tract and subtyping of the corresponding isolates.

Gills			Gastrointestinal Tract		
Sample	qPCR Assay	*Blastocystis* sp. ST	Sample	qPCR Assay	*Blastocystis* sp. ST
Wimereux					
WJ1B1	+	ST3	WJ1T1	+	ST44
WJ1B2	-		WJ1T2	+	ST3
WJ1B3	+	MI ^a^	WJ1T3	-	
WJ2B2	+	MI ^a^	WJ2T2	-	
WJ3B5	+	ST3	WJ3T5	-	
WJ4B1	+	MI ^a^	WJ4T1	+	MI ^a^
WJ4B3	-		WJ4T3	+	ST26
WJ4B7	+	MI ^a^	WJ4T7	+	ST3
WJ5B1	+	MI ^a^	WJ5T1	+	ST3
WJ5B4	-		WJ5T4	+	ST26
WJ5B5	+	ST23	WJ5T5	+	MI ^a^
WJ6B4	+	ST44	WJ6T4	-	
WJ6B12	-		WJ6T12	+	ST44
WJ7B1	+	MI ^a^	WJ7T1	+	ST3
WJ7B3	+	MI ^a^	WJ7T3	+	ST14
WJ7B4	+	ST7	WJ7T4	+	MI ^a^
WJ8B2	+	MI ^a^	WJ8T2	-	
WJ8B12	+	ST14	WJ8T12	-	
WJ9B10	+	ST3	WJ9T10	+	MI ^a^
WJ10B3	-		WJ10T3	+	MI ^a^
WJ10B4	-		WJ10T4	+	MI ^a^
WJ10B7	-		WJ10T7	+	MI ^a^
WJ10B8	-		WJ10T8	+	MI ^a^
WJ10B12	+	MI ^a^	WJ10T12	+	MI ^a^
Dannes					
CF1B2	+	MI ^a^	CF1T2	+	MI ^a^
CF1B5	-		CF1T5	+	MI ^a^
CF2B4	+	ST3	CF2T4	-	
CF5B1	+	MI ^a^	CF5T1	+	ST3
CF7B4	-		CF7T4	+	ST44
CF7B5	-		CF7T5	+	MI ^a^
CF7B6	+	MI ^a^	CF7T6	-	
CF7B7	+	MI ^a^	CF7T7	+	ST3
CF7B8	+	ST3	CF7T8	-	
CF7B9	+	ST3	CF7T9	+	ST3
CF7B10	+	ST3	CF7T10	-	
CF7B11	+	ST3	CF7T11	-	
CF7B12	+	MI ^a^	CF7T12	-	
CF8B1	+	ST3	CF8T1	+	MI ^a^
CF8B2	+	ST3	CF8T2	+	MI ^a^
CF8B3	+	MI ^a^	CF8T3	+	MI ^a^
CF8B4	+	MI ^a^	CF8T4	+	MI ^a^
CF8B5	+	MI ^a^	CF8T5	-	
CF8B6	+	MI ^a^	CF8T6	-	
CF8B7	+	MI ^a^	CF8T7	+	MI ^a^
CF8B8	+	MI ^a^	CF8T8	-	
CF8B9	+	MI ^a^	CF8T9	+	MI ^a^
CF8B10	+	MI ^a^	CF8T10	-	
CF8B11	+	MI ^a^	CF8T11	-	
CF9B1	+	ST3	CF9T1	-	
CF9B2	+	ST3	CF9T2	-	
CF9B3	+	MI ^a^	CF9T3	-	
CF9B4	+	ST3	CF9T4	-	
CF9B7	+	ST3	CF9T7	-	
CF9B8	+	ST3	CF9T8	-	
CF9B10	+	ST3	CF9T10	-	
CF9B11	+	ST3	CF9T11	-	
CF10B1	-		CF10T1	+	MI ^a^
CF10B2	-		CF10T2	+	MI ^a^
CF10B7	-		CF10T7	+	MI ^a^
CF10B9	-		CF10T9	+	MI ^a^
CF10B10	-		CF10T10	+	MI ^a^
CF10B11	-		CF10T11	+	ST44

^a^ MI, Mixed infection with unidentified STs.

**Table 3:** *Blastocystis* sp. OTUs and corresponding number of reads identified in SOMLIT marine stations samples collected at different seasons.

SOMLIT Station	OTU ^a^	Number of Reads	Sampling Date	Season	*Blastocystis* sp. ST
C (coastal)	OTU0278	202	23/07/2012	Summer	ST10a
		1	03/10/2012	Autumn	
	OTU0438	14	21/06/2012	Summer	ST3
		52	23/07/2012	Summer	
		2	13/11/2012	Winter	
L (offshore)	OTU0438	7	26/02/2013	Winter	ST3
		16	09/05/2012	Spring	
	OTU0680	2	03/10/2012	Autumn	ST2

^a^ OTU, Operational Taxonomic Unit.

## Data Availability

All relevant data are within the paper.
